# Amiodarone‐related thyroid dysfunction and associated outcomes in patients with heart failure—A nationwide cohort study

**DOI:** 10.1111/joim.70116

**Published:** 2026-05-26

**Authors:** Søren Lund Kristensen, Sam Aiyad Ali, Mads Ersbøll, Jawad Haider Butt, Rasmus Rørth, Lucas Malta Westergaard, Lauge Østergaard, Finn Gustafsson, Oren Caspi, Emil Fosbøl, Lars Køber, Christian Selmer

**Affiliations:** ^1^ Department of Cardiology Rigshospitalet University of Copenhagen Copenhagen Denmark; ^2^ Steno Diabetes Center Herlev Hospital Copenhagen Denmark; ^3^ Department of Cardiology Hvidovre Hospital Copenhagen Denmark; ^4^ Department of Cardiology Rambam Health Care Campus Haifa Israel; ^5^ Ruth and Bruce Rappaport Faculty of Medicine Technion—Israel Institute of Technology Haifa Israel; ^6^ Department of Clinical Medicine University of Copenhagen Copenhagen Denmark; ^7^ Department of Endocrinology Bispebjerg and Frederiksberg Hospital Copenhagen Denmark

**Keywords:** amiodarone, heart failure, prognosis, thyroid disorders

## Abstract

**Aim:**

Amiodarone increases the risk of thyroid dysfunction, but the prognostic significance of this complication in patients with heart failure (HF) is uncertain. This study investigates the association between amiodarone‐related thyroid dysfunction and risk of HF hospitalization or death in a nationwide cohort.

**Methods:**

Cases and controls were identified from a source population of all HF patients who initiated treatment with amiodarone from 1996 to 2021. Patients diagnosed with and/or treated for thyroid dysfunction within 3 years of starting amiodarone were classified as cases and comprised the study population together with controls matched 1:2 based on age, sex, calendar year, and HF duration. The primary outcome was a composite of HF hospitalization and death within 1 year from onset of thyroid dysfunction, analyzed using Kaplan–Meier curves and Cox regression models adjusted for comorbidities.

**Results:**

Of 21,947 HF patients who initiated amiodarone, 2972 (14%) developed thyroid dysfunction. After matching, the study population consisted of 2953 cases and 5857 controls, mean age 70 years, and 65% men. At 1‐year follow‐up the primary outcome occurred in 1154 (39%) cases and 1826 (31%) matched controls, yielding an adjusted hazard ratio (HR) of 1.30 (95% CI 1.21–1.40). This increased risk was observed for both HF hospitalization (30% vs. 23%, HR 1.35 [1.24–1.47]) and all‐cause death (19% vs. 15%, HR 1.21 [1.08–1.34]).

**Conclusions:**

In this nested case–control study derived from a nationwide cohort of amiodarone‐treated HF patients, new‐onset thyroid dysfunction was associated with a 30% higher risk of a composite of HF hospitalization and death within 1 year.

## Graphical abstract

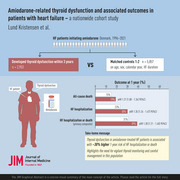

**Abbreviations**
COPD: chronic obstructive pulmonary disease
CRT: cardiac resynchronization therapy
CRT‐D: cardiac resynchronization therapy defibrillator
CRT‐P: cardiac resynchronization therapy pacemaker
HF: heart failure
HR: hazard ratio
ICD: implantable‐cardioverter defibrillator
ICD‐10: International Classification of Diseases, 10th edition
SVT: supraventricular tachycardia
VT: ventricular tachycardia

## Introduction

Arrhythmias are common in patients with heart failure (HF), and managementing of these patients is complex [1, 2]. Patients with HF have limited options for antiarrhythmic treatment, and catheter ablation procedures can be more difficult and carry a higher risk of complications [3, 4]. As a result, amiodarone is often used in patients with HF and arrythmias despite limited evidence of its efficacy in terms of improving morbidity and mortality, including findings from randomized trials comparing amiodarone and other antiarrhythmic drugs with catheter ablation in atrial fibrillation [5]. Amiodarone has a well‐recognized potential for multisystem toxicities affecting the lungs, liver, thyroid, peripheral nerves, and skin [6]. Thyroid dysfunction is frequently seen after initiation of amiodarone due to the iodine content of the drug. It is also well‐established that the presence of even subclinical hypo‐ or hyperthyroidism at the time of HF diagnosis is associated with worse prognosis [7, 8, 9]. Although this could simply reflect the severity of HF, thyroid hormones are closely related to cardiovascular function. They regulate gene expression in cardiac myocytes and can bind to ion channels on the cell and mitochondrial membranes, which, in turn, may lead to changes in cardiac contractility, vascular resistance, blood volume, and heart rate [10, 11].

However, little is known about the specific consequences of thyroid dysfunction that occurs as a result of amiodarone treatment in patients with HF, particularly regarding its risks and impact on prognosis.

Consequently, we set out to examine the prognostic impact of incident thyroid dysfunction in HF patients after initiation of amiodarone treatment.

## Methods

### Data sources

The Danish national administrative registries contain unique data on all Danish residents, and accurate linkage between multiple nationwide registries is possible using a unique and personal identification number. In the current study, data were obtained on civil status, hospital contacts, procedures, prescription fills, and mortality through linkage of the following registries: (1) the Danish Civil Registration System, which contains data on birth date, sex, and vital status [13]; (2) the Danish National Patient Registry, which holds information on all hospital admissions since 1977 and outpatient contacts since 1995, with diagnoses codes based on the International Classification of Diseases (ICD) ICD‐8 and ICD‐10 codes and surgical procedures classified according to the Nordic Medico‐Statistical Committee since 1996; (3) the Danish National Prescription Registry, which contains detailed information on dispensing date, strength, and quantity on all claimed drug prescriptions in Denmark; and (4) the Danish Registry of Cause of Death, which contains information on the date, cause, and place of death.

### Study population

**Fig. 1:**
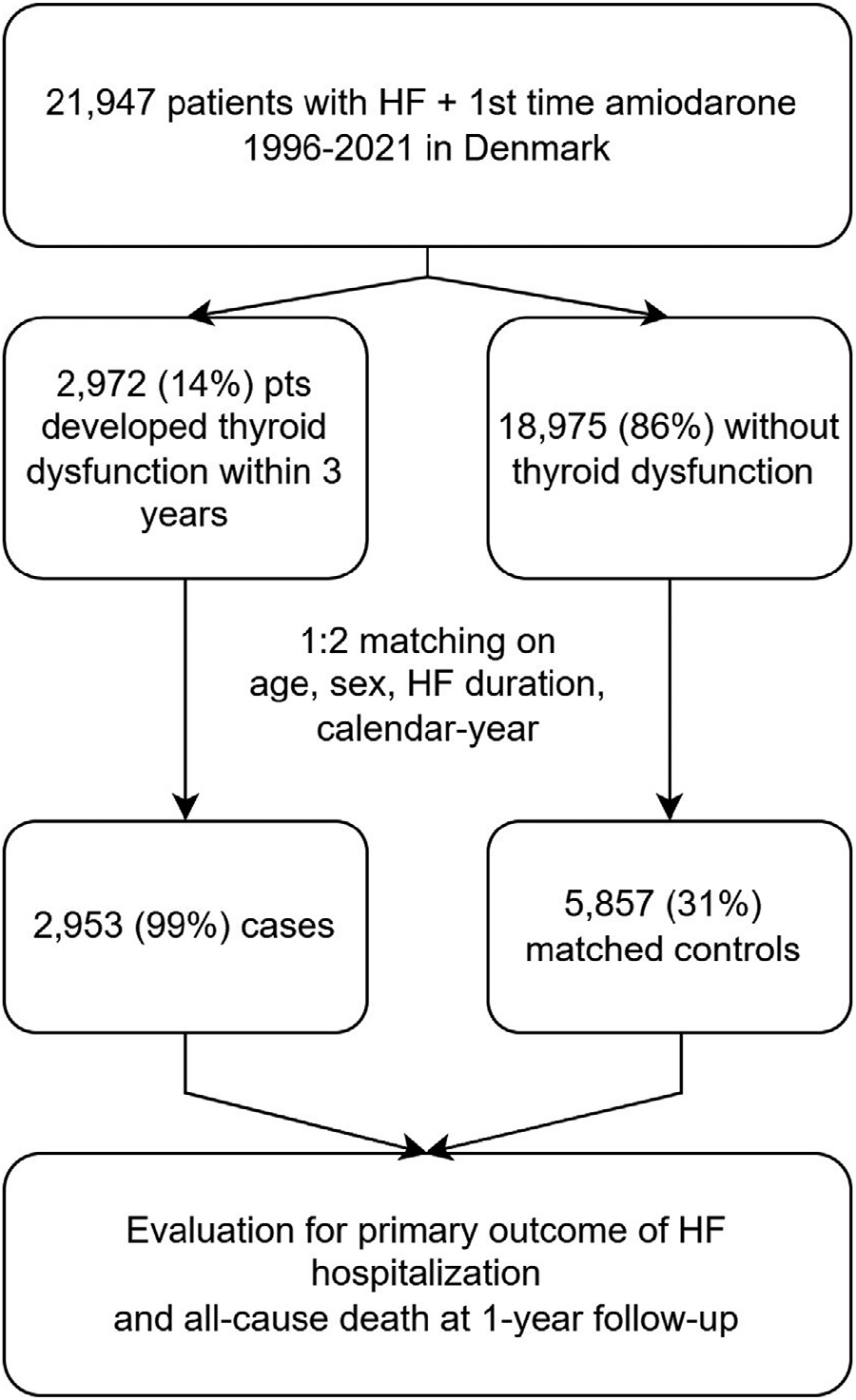
Flow chart of the study population. HF, heart failure.

The matched cases and controls were identified from a source population comprising all Danish residents aged 18–90 years who, during the study period, (1) were diagnosed with HF as either in‐ or outpatients, (2) after HF diagnosis redeemed a first prescription of amiodarone, and (3) with HF and no history of thyroid dysfunction at the time of amiodarone prescription (Fig. 1).

Cases were identified by within 3 years of amiodarone initiation to have either an in‐ or outpatient diagnosis indicating thyroid dysfunction (Table S1) or had been prescribed pharmacological treatment for thyroid dysfunction (Table S2), as thyroid function test data were not available. Table S3 shows the baseline characteristics for the source population before matching. Cases were matched 1:2 for age, sex, HF duration, and calendar year with controls sampled from the source population who did not develop thyroid dysfunction within the first 3 years after treatment initiation. The index date for cases was the date of thyroid dysfunction, whereas for matched controls, the index date was set at a similar time distance from amiodarone initiation as the case they were matched with.

### Comorbidity and concomitant pharmacotherapy

Medical history for each patient was obtained from the Danish National Patient Registry based on inhospital and outpatient diagnostic codes at any time prior to and including the index date. Concomitant pharmacotherapy was defined from the Danish National Prescription Registry as claimed prescriptions within 180 days prior to the date of a thyroid dysfunction event for cases and corresponding date set for matched controls (anatomical therapeutic classification [ATC] and ICD codes used are listed in Table S2).

### Outcomes

The primary outcome was a composite of HF hospitalization or all‐cause death at 1‐year follow‐up, and secondary outcomes were each of the components separately. The diagnosis of HF has a high accuracy in Danish registries with a specificity of 99% and a positive predictive value of 81%, albeit with a low sensitivity of 29% [12].

### Statistics

Descriptive data were reported using numbers and percentages for categorical variables and medians and interquartile ranges for continuous variables. Differences in baseline characteristics in the cases and controls were tested using the chi‐squared test for categorical variables and the Mann–Whitney test for continuous variables. Mortality was examined using Kaplan–Meier estimates, and differences between groups were assessed using the log‐rank test. The crude cumulative incidence of the composite primary outcome according to groups was estimated using the Kaplan–Meier estimator. Differences between groups were assessed with Gray's test. A multivariable‐adjusted Cox proportional hazard analysis was applied to compare risk across groups. Analysis was adjusted for the following covariates: comorbidities (prior ventricular fibrillation or cardiac arrest, ischemic heart disease, peripheral artery disease, diabetes, malignancy, chronic kidney disease, chronic obstructive pulmonary disease (COPD), and stroke) and implantable‐cardioverter defibrillator (ICD) or cardiac resynchronization therapy (CRT). Risk set matching using time from amiodarone initiation until time of thyroid dysfunction was applied to ensure that matched controls were included at a similar time point as their case counterparts.

All statistical analyses were performed using the SAS Statistical Software Version 9.4, Cary, NC, USA, and Stata Statistical Software Release 18, College Station, TX, StataCorp LLC, USA. For all analyses, a two‐tailed *p*‐value below 0.05 was considered statistically significant.

### Sensitivity analyses

In sensitivity analyses, we assessed similar endpoints for cases with indices of hypothyroidism and hyperthyroidism (and matched controls) separately, as well as reassessing the primary outcome and each of the components at 5‐year follow‐up. We also investigated how many patients were identified as having thyroid dysfunction during an HF hospitalization or within 7 days thereafter.

Finally, we assessed whether outcomes and prognostic importance of thyroid dysfunction differed according to history of ventricular arrhythmia.

### Ethics

This study was approved by the Capital Region of Denmark (approval number: P‐2019‐348) in accordance with the General Data Protection Regulation. In Denmark, there is no requirement for ethics committee approval in registry‐based studies in which individuals cannot be identified.

## Results

### Study population and baseline characteristics

The source population comprised 21,947 subjects with HF, no history of thyroid dysfunction, who initiated treatment with amiodarone. A total of 2972 subjects (14%) developed thyroid dysfunction, and the study population was derived based on matching these cases 1:2 with subjects from the source population who did not develop thyroid dysfunction. After matching, the study population comprised 2953 cases with thyroid dysfunction at baseline, of whom 1372 (46%) had hyperthyroidism and 1581 (54%) had hypothyroidism, and 5857 matched controls. No matched controls were available for remaining 19 (0.6%) cases who were, therefore, excluded from the study population, Fig. 1. Baseline characteristics of the study population are depicted in Table 1; mean age was 70 years, and 65% were men. Mean time from HF diagnosis to amiodarone initiation was 4.0 years, and most cases (62%) were included based on a diagnosis of thyroid dysfunction (and most likely filled in a prescription later), whereas remaining 38% were included based on a filled prescription of thyroid medication. A history of atrial fibrillation (72% vs. 70%, *p* = 0.011), ventricular tachycardia (VT) (23% vs. 19%, *p* < 0.001), and prior implantation of an ICD or CRT defibrillator (CRT‐D) (28% vs. 23%, *p* < 0.001) were more prevalent among cases than matched controls.

**Table 1:** Baseline characteristics of the study population after matching.

	Thyroid cases	Matched controls	*p*‐value
*N*	2953	5857	
Age, years, and mean (SD)	70.1 (11.1)	70.1 (10.9)	0.69
Male sex	1916 (65%)	3823 (65%)	
HF duration, years, and mean (SD)	4.0 (4.4)	3.9 (4.4)	0.56
Calendar period			0.99
1996–2002	430 (14%)	843 (14%)	
2003–2008	675 (23%)	1352 (23%)	
2009–2015	938 (32%)	1858 (32%)	
2016–2021	910 (31%)	1804 (31%)	
Time from amiodarone initiation to study inclusion (thyroid dysfunction)			
<90 days	497 (17%)	NA	
3 months–1 year	1197 (40%)	NA	
>1–3 years	1259 (43%)	NA	
Study inclusion based on			
Indices of hyperthyroidism	1372 (46%)	NA	
Indices of hypothyroidism	1581 (54%)	NA	
Ischemic heart disease	1127 (38%)	2211 (38%)	0.70
Atrial fibrillation	2129 (72%)	4069 (70%)	0.011
Unspecified supraventricular tachycardia	302 (10%)	637 (11%)	0.82
Ventricular tachycardia	671 (23%)	1091 (19%)	<0.001
Hypertension	970 (33%)	2044 (35%)	0.064
Stroke	291 (10%)	524 (9%)	0.17
Peripheral artery disease	204 (7%)	374 (6%)	0.35
Diabetes	433 (15%)	804 (14%)	0.23
COPD	550 (19%)	903 (15%)	<0.001
Malignancy	274 (9%)	547 (9%)	0.93
Liver disease	50 (2%)	96 (2%)	0.85
Rheumatic disease	80 (3%)	130 (2%)	0.16
Chronic kidney disease	375 (13%)	616 (11%)	0.002
ICD or CRT‐D	828 (28%)	1347 (23%)	<0.001
CRT‐P	82 (3%)	139 (2%)	0.25
**Pharmacotherapy**			
Beta blocker	2041 (69%)	4074 (70%)	0.67
CCB	421 (14%)	924 (16%)	0.061
RASi (ACE‐i, ARB, and ARNI)	2196 (74%)	4272 (73%)	0.15
Thiazide	329 (11%)	670 (11%)	0.68
Loop diuretic	2223 (75%)	3982 (68%)	<0.001
MRA	990 (34%)	1804 (31%)	0.009
Digoxin	714 (24%)	1376 (24%)	0.48
Statin	1433 (49%)	2765 (47%)	0.24
Oral glucose lowering agent	504 (17%)	942 (16%)	0.24
NSAID	285 (10%)	577 (10%)	0.77
Acetylsalicyclic acid	1264 (43%)	2433 (42%)	0.26
OAC	1803 (61%)	3560 (61%)	0.80

Abbreviations: CCB, calcium channel blocker; COPD, chronic obstructive pulmonary disease; CRT‐D/P, cardiac resynchronization therapy defibrillator/pacemaker; HF, heart failure; ICD, implantable‐cardioverter defibrillator; IHD, ischemic heart disease; MRA, mineralocorticoid receptor antagonist; NSAID, nonsteroidal anti‐inflammatory drug; OAC, oral anticoagulant; RASi, renin angiotensin system inhibitor.

In contrast, a history of ischemic heart disease (38% vs. 38%), stroke (10% vs. 9%), and diabetes (15% vs. 14%) were distributed similarly in the two groups, whereas COPD (19% vs. 15%) and chronic kidney disease (13% vs. 11%) were more prevalent among cases. In terms of HF medication, cases were more likely to be on loop diuretics (75% vs. 68%) and mineralocorticoid receptor antagonist (34% vs. 31%), whereas other cardiovascular and non‐cardiovascular pharmacotherapies were evenly used across the two groups.

### Outcomes

**Fig. 2:**
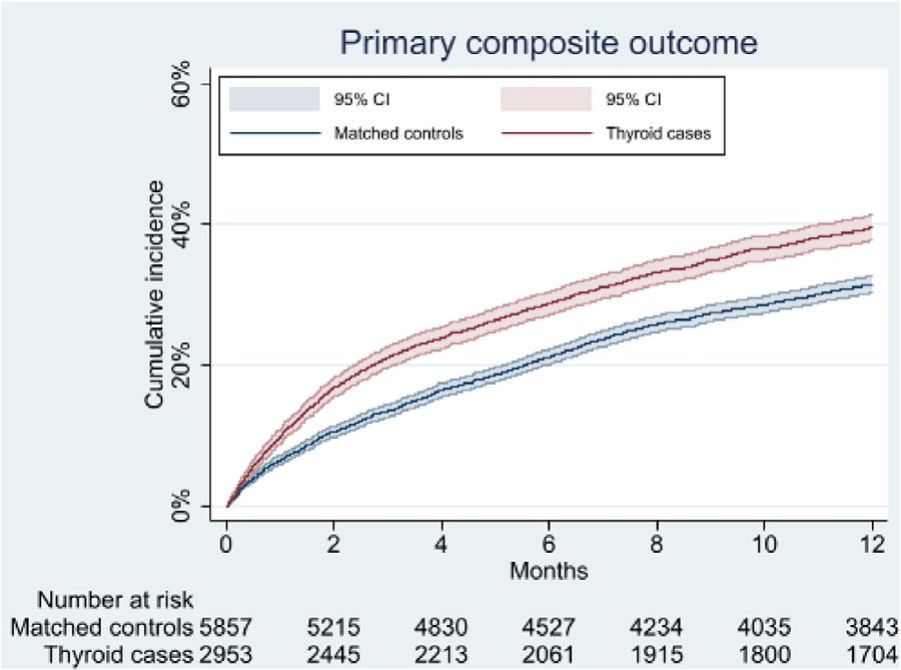
1‐year risk of the primary composite endpoint of heart failure (HF) hospitalization or all‐cause death.

**Fig. 3:**
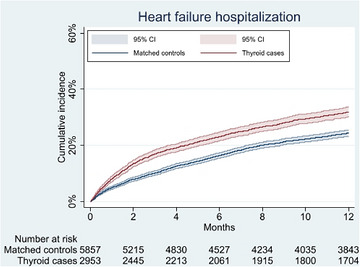
1‐year risk of heart failure (HF) hospitalization.

**Fig. 4:**
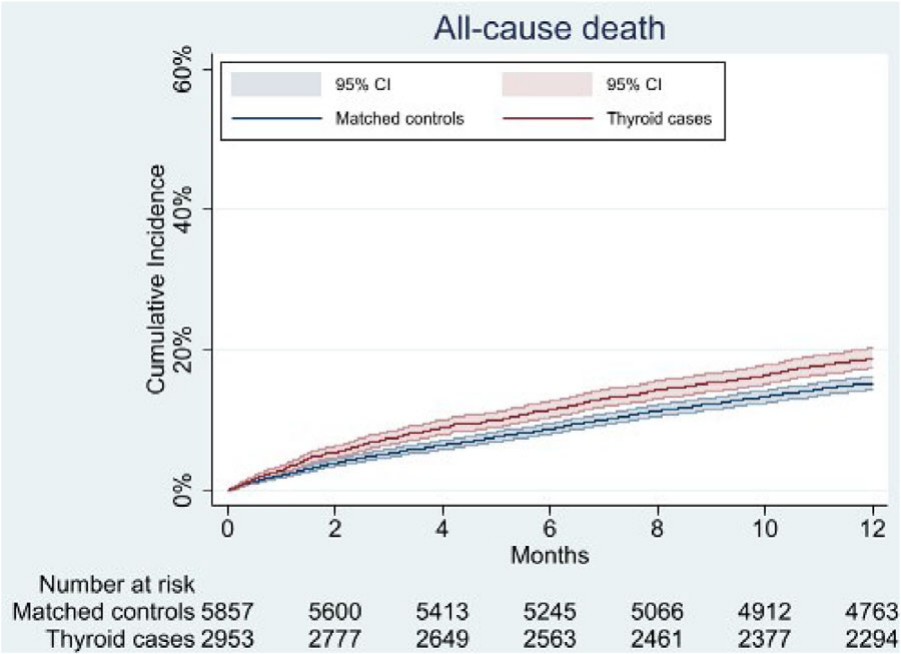
1‐year risk of all‐cause death.

The primary composite outcome of HF hospitalization or all‐cause death at 1‐year follow‐up occurred in 1154 (39%) cases versus 1826 (31%) matched controls, corresponding to an adjusted hazard ratio (HR) of 1.30 (95% CI 1.21–1.40) with matched controls as reference (Fig. 2). HF hospitalization was the most frequently occurring of the two components of the primary endpoint with 886 (30%) events among cases versus 1342 (23%) among matched controls; adjusted HR 1.37 (95% CI 1.25–1.49), Fig. 3. All‐cause death at 1‐year follow‐up occurred in 545 (19%) of cases versus 875 (15%) among matched controls, yielding an adjusted HR of 1.21 (95% CI 1.08–1.34), Fig. 4. At 5‐year follow‐up, the primary outcome occurred in 1962 (66%) of cases versus 3487 (60%) of matched controls, giving an HR of 1.20 (95% CI 1.13–1.27), Table S4.

When examining thyroid dysfunction as hypo‐ and hyperthyroidism separately, we found that at 1‐year follow‐up event rates of the primary outcome were numerically higher among patients with hypothyroidism versus those with hyperthyroidism (58.1 vs. 50.5 events per 100 patient‐years), primarily driven by differences in all‐cause death rates (24.7 vs. 17.1 per 100 patient‐years). However, compared to their matched controls, both cases of hypo‐ and hyperthyroidism were associated with higher risk of the primary composite endpoint (HR 1.34 [95% CI 1.20–1.50] and HR 1.24 [95% CI 1.13–1.37], respectively; see Table 2). At 5‐year follow‐up, the relative risk associated with thyroid dysfunction was somewhat attenuated. In particular, the risk associated with hyperthyroidism was no longer associated with an excess risk of all‐cause death (Table S4). A total of 163 of cases were identified as having thyroid dysfunction in relation to or within a week after an HF hospitalization, and these patients were at a more than two‐fold increased risk of the primary composite outcome.

**Table 2:** 1‐year outcomes after amiodarone‐related thyroid dysfunction.

	No. events/No. patients	Crude rate per 100 py (95% CI)	Adjusted HR (95% CI)	*p*‐value
**Primary composite outcome**				
**Overall**				
Thyroid cases	1154/2953	54.5 (51.4–57.7)	1.30 (1.21–1.40)	<0.001
Matched controls	1826/5857	39.3 (37.6–41.2)	1.00 (ref)	
**Subgroups**				
Hyperthyroid cases	498/1372	50.5 (46.2–55.1)	1.24 (1.13–1.37)	<0.001
Matched controls	771/2718	44.0 (33.4–38.5)	1.00 (ref)	
Hypothyroid cases	656/1581	58.1 (53.8–63.0)	1.34 (1.19–1.50)	<0.001
Matched controls	1034/3139	42.4 (39.9–45.1)	1.00 (ref)	
**HF hospitalization**				
**Overall**				
Thyroid cases	886/2953	41.8 (39.1–44.7)	1.35 (1.24–1.47)	<0.001
Matched controls	1342/5857	28.9 (27.6–30.8)	1.00 (ref)	
**Subgroups**				
Hyperthyroid	396/1372	40.0 (36.3–44.2)	1.39 (1.22–1.58)	<0.001
Matched controls	596/2718	27.5 (25.4–29.8)	1.00 (ref)	
Hypothyroid	490/1581	43.3 (39.7–47.4)	1.32 (1.17–1.48)	<0.001
Matched controls	746/3139	30.6 (28.5–32.9)	1.00 (ref)	
**All‐cause death**				
**Overall**				
Thyroid dysfunction	545/2953	21.2 (19.5–23.0)	1.21 (1.08–1.34)	<0.001
Matched controls	875/5857	18.3 (17.1–19.5)	1.00 (ref)	
**Subgroups**				
Hyperthyroid	206/1372	17.1 (14.9–19.6)	1.22 (1.02–1.45)	0.028
Matched controls	356/2718	14.7 (13.2–16.3)	1.00 (ref)	
Hypothyroid	338/1581	24.7 (22.2–27.5)	1.19 (1.04–1.36)	0.013
Matched controls	588/3319	21.4 (19.8–23.2)	1.00 (ref)	

*Note*: Adjusted for comorbidities (prior ventricular fibrillation or cardiac arrest, ischemic heart disease, peripheral artery disease, diabetes, malignancy, chronic kidney disease, chronic obstructive pulmonary disease, and stroke) and implantable‐cardioverter defibrillator (ICD) or cardiac resynchronization therapy (CRT).

Abbreviation: HR, hazard ratio.

The primary outcome at 1‐year occurred more often in patients with a history of ventricular arrythmia at baseline (47% of cases and 41% of controls), whereas we found a similar increased risk associated with thyroid dysfunction in those with and without a history of ventricular arrhythmia (data not shown).

## Discussion

In the present study, we examined whether the development of amiodarone‐related thyroid dysfunction was associated with worse outcomes in patients with HF.

Our main findings were as follows: (1) The high‐risk nature of the amiodarone‐treated HF patients studied was confirmed with a 1‐year incidence of the composite outcome approaching 40% and an all‐cause mortality around 20%. (2) The development of thyroid dysfunction was associated with around 30% higher 1‐year risk of the composite endpoint, primarily driven by an excess risk of HF hospitalization but also a significantly increased risk of all‐cause death. (3) Similar risk patterns were observed for both hypo‐ and hyperthyroidism, with no significant differences in relative risk compared to their respective controls, albeit the absolute risk of events was higher in patients with hypothyroidism. When assessing long‐term outcomes, the adverse cardiovascular risk associated with thyroid dysfunction was lower. Interestingly, it seemed there was a more transient association to outcomes for patients with hyperthyroidism, in the sense that it was no longer associated with excess mortality at 5‐year follow‐up, whereas the excess mortality risk persisted for patients with hypothyroidism when compared to the matched controls.

Another important aspect of our study was that 38% of cases were first identified on the basis of a filled prescription of thyroid medication rather than a diagnosis, indicating that in many of these cases treatment was initiated in primary care.

The role of amiodarone in treatment of patients with HF is somewhat controversial, and despite proven clinical benefits on reducing ventricular arrhythmia burden, amiodarone did not improve survival versus placebo, it was inferior in comparison with ICD implantation for prevention of sudden cardiac death, and further in a post hoc analysis of the COMET trial, amiodarone use was associated with worse outcomes [13, 14, 15].

One interesting additional finding of our study was that around 5% of the cases with thyroid dysfunction was diagnosed in relation to or shortly after an HF hospitalization, indicating that their thyroid dysfunction may have played a role in those events preceding the studied time period, and thus, we may be underestimating the importance of thyroid dysfunction as these events are not counted in the present analysis.

Thyroid dysfunction is well recognized as a contributing factor in the development of HF and is linked to poorer prognosis. Although hypothyroidism is more frequently associated with HF, hyperthyroidism tends to precipitate a more severe decompensation [16, 17, 18, 19].

However, there are limited data specifically addressing amiodarone‐related thyroid dysfunction in patients with HF. Retrospective studies on patients with cardiac arrhythmia (not restricted to those with HF) on amiodarone therapy have reported a high risk of thyroid‐related adverse events but did not include comparator groups. This raises the possibility that these patients are at high risk even in the absence of thyroid dysfunction [7, 20, 21].

European guidelines recommend treating amiodarone‐induced thyrotoxicosis and considering the discontinuation of amiodarone, whereas for amiodarone‐induced hypothyroidism, they recommend levothyroxine (LT4) substitution and amiodarone continuation [22, 23]. A more conservative approach is recommended for patients with subclinical hypothyroidism based on a study indicating no benefit from substitution in elderly patients without HF [24].

It is important to underline the scope of the present manuscript, which is to assess the impact of amiodarone‐related thyroid dysfunction on risk of worsening HF or death and not to evaluate the role of amiodarone dosing nor subsequent management strategies, including amiodarone discontinuation. As such, our study touches upon an important clinical dilemma. Should physicians accept and potentially treat thyroid dysfunction, or should they discontinue amiodarone with the potential risk of an increased burden of arrhythmias for the patient?

Although we cannot definitely answer these questions due to the inherent limitations of the observational design, our findings underline the high risk of serious adverse events in HF patients with amiodarone‐related thyroid dysfunction, and that the emergence of thyroid dysfunction in this setting should lead to careful consideration of treatment options. This may include referrals for advanced HF therapies such as left ventricular assist device or heart transplantation, treatment options, which, in turn, would necessitate close monitoring and management of thyroid dysfunction.

### Strengths and limitations

The current study has several strengths. It is based on a large nationwide cohort of unselected patients, and , amiodarone is primarily prescribed by cardiologists meaning that most patients are followed in a specialized hospital setting, which helps minimize the risk of surveillance bias. There are also major limitations, which include lack of information on thyroid‐stimulating hormone, free thyroid hormones, thyroid antibodies, and thyroid scintigraphy tests, which meant we were not able to define hypo‐ and hyperthyroidism biochemically nor define and distinct underlying causes. This information would allow for a more accurate classification of thyroid dysfunction and help determine if severity of thyroid disease correlates with cardiovascular outcomes and death. We did not have information on primary care diagnoses and thus may have missed cases conservatively managed in this setting.

We did not include prednisolone and cholestyramine in our list of antithyroid medications due to their widespread use for other conditions. Furthermore, we lack important clinical information related to HF status, such as left ventricular ejection fraction, systolic blood pressure, New York Heart Association function class and N‐terminal pro b‐type natriuretic peptide levels. We did not have information on dosing regimen of amiodarone, which likely is related to disease severity and neither timing nor reasons for discontinuations. Finally, patients with thyroid dysfunction had higher rates of loop diuretic use, COPD, VT, and ICD or CRT‐D implantation, which may have contributed to the worse outcomes observed in this group.

## Conclusion

In this nested case–control study derived from a nationwide cohort of amiodarone‐treated HF patients, the development of thyroid dysfunction was associated with a 30% increased risk of experiencing a composite outcome of HF hospitalization or all‐cause death within 1 year. This study highlights the prognostic significance of thyroid dysfunction among high‐risk HF patients recently treated with amiodarone.

## Author contributions

**Rasmus Rørth**: Conceptualization; writing—review and editing; methodology. **Lucas Malta Westergaard**: Writing—review and editing; formal analysis. **Søren Lund Kristensen**: Conceptualization; methodology; writing—review and editing; writing—original draft. **Finn Gustafsson**: Conceptualization; writing—review and editing; supervision. **Sam Aiyad Ali**: Conceptualization; methodology; formal analysis. **Jawad Haider Butt**: Writing—review and editing; data curation; supervision. **Mads Ersbøll**: Conceptualization; writing—review and editing. **Lauge Østergaard**: Writing—review and editing; methodology; supervision. **Oren Caspi**: Conceptualization; writing—review and editing; validation; methodology; formal analysis; project administration. **Lars Køber**: Supervision; writing—review and editing; validation; methodology. **Christian Selmer**: Conceptualization; investigation; writing—original draft; validation; visualization; writing—review and editing; formal analysis; project administration; supervision; resources. **Emil Fosbøl**: Writing—review and editing; validation; methodology; supervision.

## Conflict of interest statement

The authors declare no conflicts of interest.

## Funding information

No sources of funding were available for this study.

## Supporting information




**Table S1**: ICD‐8 and ICD‐10 classification codes for medical diagnoses and classification codes for cardiac procedures.
**Table S2**: Classification codes for pharmacotherapy.
**Table S3**: Baseline of study population prior to matching.
**Table S4**: 5‐year outcomes after amiodarone‐related thyroid dysfunction.
**Figure S1**: Primary outcome of HF hospitalization or death at 5‐year follow‐up.
**Figure S2**: HF hospitalization at 5‐year follow‐up.
**Figure S3**: All‐cause death at 5‐year follow‐up.
