# Supplementary material for: Amiodarone‐related thyroid dysfunction and associated outcomes in patients with heart failure—A nationwide cohort study
Source: J Intern Med. 2026 May 26;300(2):204–12. doi: 10.1111/joim.70116 (PMC13327501; doi:10.1111/joim.70116)

**APPENDIX**

Supplementary Table 1: ICD-8 and ICD-10 Classification codes for medical diagnoses and classification codes for cardiac procedures

| **Comorbidity** | **ICD-8 code(s)** | **ICD-10 code(s)** |
| --- | --- | --- |
| Thyroid Disease,  Congenital Iodine—Deficiency Syndrome, Iodine-deficiency-related thyroid disorders, Subclinical iodine-deficiency hypothyroidism, Hypothyroidism  Nontoxic Goitre, Thyrotoxicosis  Thyroiditis, Unspecified thyroid disorders | NA | E00, E01, E02. E03. E04, E05. E06. E07 |
| Atrial fibrillation | 42793, 42794 | I48 |
| Supraventricular Tachycardia |  | I471 |
| Ventricular Tachycardia |  | I470, I472 |
| Ventricular fibrillation |  | I490 |
| Cardiac Arrest |  | I46 |
| Unspecified Arrhythmias |  | I49, I479 |
| Stroke | Occlusion of precerebral arteries: 432,  Cerebral thrombosis: 433,  Cerebral embolism: 434,  Transient ischemic attach: 435,  Acute but ill-defined cerebrovascular disease: 436 | Ischemic stroke: I63  Unspecified stroke: I64  Transient ischemic attach: G45 |
| Ischemic Heart Disease | 410-414 | I20-I25 |
| Peripheral vascular disease | 440, 444 | I70, I74 |
| Heart failure | 425, 428, 42709, 42710, 42711, 42719,42899 | I42, I43, I50, I099A, I110, I130, I132, I255, J819 |
| Chronic kidney disease | 403-404, 581-584, 25002, 40039, 59009, 59320, 75310, 75311, 75319  DM400, DM313, DM319, DM321B | N02-N08, N11-N14, N18-N19, N26,  Q61, N158, N159, E102, E112, E132, E142, I120, N160, N162-N164, N168, Z992, |
| Acute Renal Insufficiency | 584 | N17, N19, R34 |
| Diabetes | 250 | E10-E14 |
| Chronic obstructive pulmonary disease | 490-492, 515-518 | J42, J43, J44 |
| Liver disease | 070, 155, 571-573 | B15-B19, C22, D684C, K70-K77, I982B, Q618A, Z944 |
| Malignancy | 140-207 | C00-C97 |
| Hypertension |  | I10 |
|  | Procedure codes |  |
| Implantable cardioverter-defibrillator (ICD) | BFCB0 |  |
| Cardiac resynchronization therapy defibrillator (CRT-D) | BFCA03 |  |
| Cardiac resynchronization therapy pacemaker (CRT-P) | BFCA04-6 |  |

Supplementary Table 2: Classification codes for pharmacotherapy

| Pharmacotherapy | ATC Code(s) |
| --- | --- |
| **Thyroid medications:** |  |
| Levothyroxine | H03AA01 |
| Liothyronine | H03AA02 |
| Thyroid | H03AA05 |
| Propylthiouracil | H03BA02 |
| Methimazole | H03BB01, H03BB02 |
| **Other medications** |  |
| Anti-diabetic medication | A10 |
| Statins | C10AA |
| Beta-blockers | C07, C09BX |
| Calcium Channel Blockers | C07F, C08, C09BB, C09DB |
| Renin-angiotensin-system inhibitors | C09 |
| Vasodilator Drugs | C02DB, C02DD, C02DG |
| Antiadrenergic Drugs | C02A, CO2B, CO2C |
| Thiazides | C03A, C07B, C07D, C02DA, C09XA52, C03EA01 |
| Loop Diuretics | C03C, C03EB01, C03EB02 |
| Mineralocorticoid Receptor Antagonists | C03DA01, C03DA02, C03DA03, C03DA04 |
| Digoxin | C01A |
| Aspirin | BO1AC06, N02BA01 |
| Vitamin K antagonists | B01AA03, B01AA04 |
| Oral anticoagulant therapy | B01AE07, B01AF01, B01AF02, B01AF03 |
| ATC: Anatomical Therapeutic Chemical | |

Supplementary Table 3 – Baseline of study population prior to matching

|  | Incident thyroid dysfunction | No incident thyroid dysfunction | P-value |
| --- | --- | --- | --- |
| N | 2,972 | 18,975 |  |
| Age, mean (SD) | 70 (11) | 70 (11) | 0.58 |
| Male sex | 1,922 (65%) | 14,324 (76%) | <0.001 |
| HF duration | 2.9 (4.4) | 2.9 (4.6) | 0.45 |
| Calendar Period |  |  | <0.001 |
| 1996-2002 | 579 (20%) | 3,312 (18%) |  |
| 2003-2008 | 684 (23%) | 4,174 (22%) |  |
| 2009-2015 | 977 (33%) | 5,522 (29%) |  |
| 2016-2021 | 732 (24%) | 5,967 (31%) |  |
| Time from amiodarone initiation to study inclusion (thyroid dysfunction) |  |  |  |
| <90 days | 498 (17%) | ~~NA~~ |  |
| 3 months-1 year | 1,200 (40%) | ~~NA~~ |  |
| >1-3 years | 1,274 (43%) | ~~NA~~ |  |
| Study inclusion based on |  |  |  |
| Indices of hyperthyroidism | 1,388 (47%) | NA |  |
| Indices of hypothyroidism | 1,584 (53%) | NA |  |
| **Medical History** |  |  |  |
| Ischemic heart disease | 1080 (36%) | 6664 (35%) | 0.20 |
| Atrial fibrillation | 2046 (69%) | 12650 (67%) | 0.019 |
| Unspecified SVT | 258 (9%) | 1515 (8%) | 0.19 |
| Ventricular tachycardia | 616 (21%) | 3295 (17%) | <0.001 |
| Hypertension | 876 (30%) | 5747 (30%) | 0.37 |
| Stroke | 265 (9%) | 1591 (8%) | 0.33 |
| Peripheral artery disease | 173 (6%) | 1196 (6%) | 0.31 |
| Diabetes | 396 (13%) | 2556 (14%) | 0.83 |
| COPD | 469 (16%) | 2859 (15%) | 0.31 |
| Malignancy | 234 (8%) | 1813 (10%) | 0.003 |
| Liver disease | 38 (1%) | 329 (2%) | 0.072 |
| Rheumatic disease | 77 (3%) | 441 (2%) | 0.37 |
| Chronic kidney disease | 245 (8%) | 1711 (9%) |  |
| **Cardiac devices** |  |  |  |
| ICD or CRT-D | 709 (24%) | 3751 (20%) | <0.001 |
| CRT-P | 52 (2%) | 321 (2%) | 0.82 |
| **Pharmacotherapy** |  |  |  |
| Beta blocker | 1872 (63%) | 11877 (63%) | 0.68 |
| Calcíum channel blocker | 646 (22%) | 3997 (21%) | 0.40 |
| RASi | 1874 (63%) | 11917 (63%) | 0.79 |
| Thiazide | 425 (14%) | 2453 (13%) | 0.039 |
| Loop diuretic | 1672 (56%) | 10519 (55%) | 0.40 |
| MRA | 671 (23%) | 4291 (23%) | 0.96 |
| Digoxin | 166 (6%) | 1050 (6%) | 0.91 |
| Statin | 861 (29%) | 5081 (27%) | 0.012 |
| Oral gluc. lowering agent | 1231 (41%) | 7779 (41%) | 0.66 |
| NSAID | 463 (16%) | 3148 (17%) | 0.17 |
| Acetylsalicyclic acid | 388 (13%) | 2473 (13%) | 0.97 |
| OAC | 259 (9%) | 1756 (9%) | 0.34 |

CCB – calcium channel blocker, COPD – chronic obstructive pulmonary disease, CRT-D/P cardiac resynchronization therapy defibrillator/pacemaker, HF – heart failure, ICD – impantable cardioverter defibrillator, IHD – Ischemic heart disease, MRA – Mineralocorticoid receptor antagonists, NSAID – non-steroidal antiinflammatory drugs, OAC – oral anticoagulants, RASi, Renin angiotensin system inhibitors, SVT – supraventricular tachycardia,

Supplementary Table 4: 5-year outcomes after amiodarone-related thyroid dysfunction

|  | No. events/ no patients | Crude rate per 100 py | Adjusted HR* | P-value |
| --- | --- | --- | --- | --- |
|  |  |  |  |  |
| **Primary Composite** |  |  |  |  |
| **Overall** |  |  |  |  |
| Thyroid cases | 1,962/2,953 | 31.0 (29.7-32.4) | 1.20 (1.13-1.27) | <0.001 |
| Matched controls | 3,487/5,857 | 24.1 (23.3-24.9) |  |  |
| **Subgroups** |  |  |  |  |
| Hyperthyroidism | 827/1,372 | 26.6 (24.8-28.5) | 1.16 (1.06-1.26) | 0.001 |
| Matched controls | 1,532/2,718 | 22.2 (21.1-23.3) |  |  |
| Hypothyroidism | 1,135/1,581 | 35.3 (33.3-37.4) | 1.23 (1.14-1.33) | <0.001 |
| Matched controls | 1,955/3,139 | 25.9 (24.8-27.0) |  |  |
|  |  |  |  |  |
| **HF hospitalization** |  |  |  |  |
| **Overall** |  |  |  |  |
| Thyroid cases | 1,437/2,953 | 22.7 (21.6-23.9) | 1.24 (1.17-1.33) | <0.001 |
| Matched controls | 2,455/5,857 | 17.0 (16.3-17.7) |  |  |
| **Subgroups** |  |  |  |  |
| Hyperthyroidism | 631/1,372 | 20.3 (18.8-21.9) | 1.20 (1.09-1.32) | <0.001 |
| Matched controls | 1,129/2,718 | 16.3 (15.4-17.3) |  |  |
| Hypothyroidism | 806/1,581 | 25.1 (23.4-26.9) | 1.29 (1.18-1.41) | <0.001 |
| Matched controls | 1,326/3,139 | 17.5 (16.6-18.5) |  |  |
| **All-cause death** |  |  |  |  |
| **Overall** |  |  |  |  |
| Thyroid dysfunction | 1,312/2,953 | 14.3 (13.5-15.1) | 1.12 (1.05-1.20) | 0.001 |
| Matched controls | 2,335/5,857 | 12.1 (11.7-12.6) |  |  |
| **Subgroups** |  |  |  |  |
| Hyperthyroidism | 493/1,372 | 11.0 (10.1-12.0) | 1.07 (0.96-1.19) | 0.247 |
| Matched controls | 940/2,718 | 10.2 (9.5-10.8) |  |  |
| Hypothyroidism | 819/1,581 | 17.4 (16.3-18.7) | 1.16 (1.06-1.26) | 0.001 |
| Matched controls | 1,395/3,319 | 13.9 (13.2-14.7) |  |  |

*Adjusted for comorbidities (prior ventricular fibrillation or cardiac arrest, ischaemic heart disease, peripheral artery disease, diabetes, malignancy, chronic kidney disease, chronic obstructive pulmonary disease, and stroke) and implantable cardioverter defibrillator (ICD) or cardiac resynchronization therapy (CRT)

Supplementary Figure 1: Primary outcome of HF hospitalization or death at 5-year follow-up


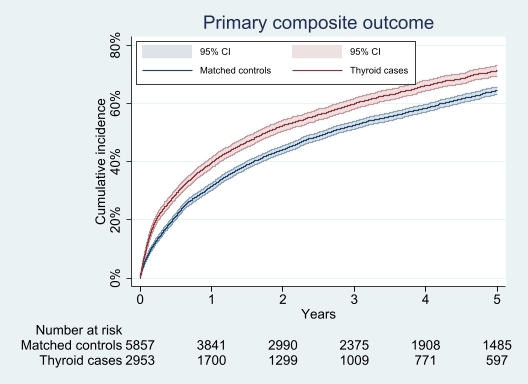


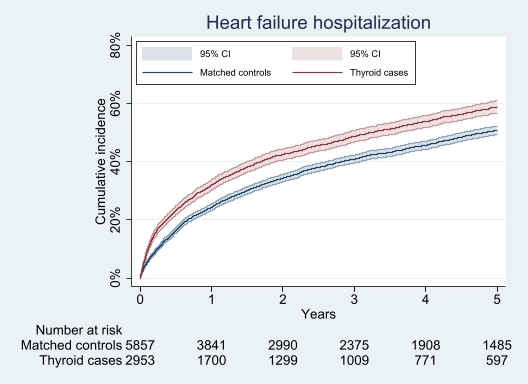
Supplementary Figure 2: HF hospitalization at 5-year follow-up

Supplementary Figure 3: All-cause death at 5-year follow-up


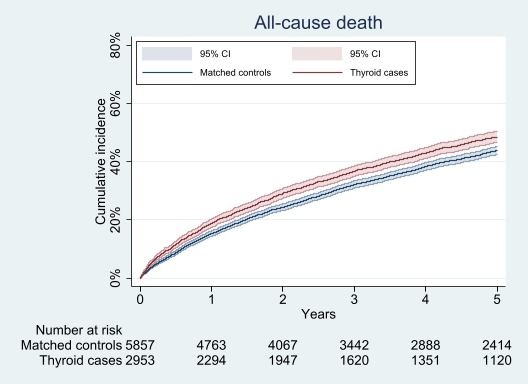

Supplement: Supplementary file 1 — Table S1: ICD‐8 and ICD‐10 classification codes for medical diagnoses and classification codes for cardiac procedures. Table S2: Classification codes for pharmacotherapy. Table S3: Baseline of study population prior to matching. Table S4: 5‐year outcomes after amiodarone‐related thyroid dysfunction. Figure S1: Primary outcome of HF hospitalization or death at 5‐year follow‐up. Figure S2: HF hospitalization at 5‐year follow‐up. Figure S3: All‐cause death at 5‐year follow‐up. [file JOIM-300-204-s001.docx]
